# Total and Unbound Pharmacokinetics of Cefiderocol in Critically Ill Patients

**DOI:** 10.3390/pharmaceutics14122786

**Published:** 2022-12-13

**Authors:** Noël Zahr, Saik Urien, Benoit Llopis, Gaëlle Noé, Nadine Tissot, Kevin Bihan, Helga Junot, Clémence Marin, Bochra Mansour, Charles-Edouard Luyt, Alexandre Bleibtreu, Christian Funck-Brentano

**Affiliations:** 1 Pharmacokinetics and Therapeutic Drug Monitoring Unit, Department of Pharmacology, Pitié-Salpêtrière Hospital, Inserm, CIC-1901, UMR-S 1166, AP-HP Sorbonne Université, 75013 Paris, France; 2 Unité de Recherche Clinique, Hôpital Necker-Enfants Malades, AP-HP, 75015 Paris, France; 3 Pharmacy Department, Pitié-Salpêtrière Hospital, AP-HP Sorbonne Université, 75013 Paris, France; 4 Service de Médecine Intensive Réanimation, Institut de Cardiologie, AP-HP Sorbonne-Université, Pitié-Salpêtrière Hospital, 75013 Paris, France; 5 Service de Maladies Infectieuses et Tropicales, Pitié-Salpêtrière Hospital, AP-HP Sorbonne Université, 75013 Paris, France

**Keywords:** cefiderocol, pharmacokinetics, PK/PD, antibiotics, drug monitoring

## Abstract

Background: Cefiderocol is a siderophore cephalosporin antibiotic active against Gram-negative bacteria, including extended-spectrum beta-lactamase and carbapenemase-producing strains. The pharmacokinetics of cefiderocol has been studied in healthy subjects and particularly in phase II and III studies. This retrospective study investigated intravenous cefiderocol population pharmacokinetics in adult patients treated by cefiderocol. Methods: We studied 55 consecutive patients hospitalized in an intensive care unit. Cefiderocol plasma samples were obtained on different occasions during treatment. Plasma concentration was assayed using mass spectrometry. Data analysis was performed using a non-linear mixed-effect approach via Monolix 2020R1. Results: A total of 205 plasma samples were obtained from 55 patients. Eighty percent of patients received cefiderocol for ventilator-associated pneumonia due to carbapenem-resistant *Pseudomonas aeruginosa* infection. Cefiderocol concentration time-courses were best fit to a two-compartment open model with first-order elimination. Elimination clearance was positively related to renal function (estimated by the CKD formula). Adding albumin plasma binding in the model significantly improved the model assuming a ~40% unbound drug fraction given a ~40 g/L albuminemia. The final model included CKD plus cefiderocol plasma binding effects. Fat-free mass was better than total body weight to influence, via the allometric rule, clearance and volume terms, but this effect was negligible. The final clearance based on free circulating drug (CL_U_) for a typical patient, CKD = 90, was 7.38 L/h [relative standard error, RSE, 22%] with a between-subject variability of 0.47 [RSE 10%] (exponential distribution). Conclusion: This study showed that albumin binding and CKD effects were significant predictors of unbound and total plasma cefiderocol concentrations. Our results indicate that individual adjustment of cefiderocol can be used to reach high minimum inhibitory concentrations based on an estimation of unbound drug concentration and optimize therapeutic efficacy.

## 1. Introduction

Cefiderocol is a cephalosporin active against most Gram-negative bacteria, including extended-spectrum beta-lactamase and carbapenemase-producing strains such as *Pseudomonas aeruginosa*, *Acinetobacter baumannii* and *Enterobacteriales* [1]. Cefiderocol was approved by the U.S. Food and Drug Administration and the European Medicines Agency for the treatment of complicated urinary tract infections, the treatment of hospital-acquired bacterial pneumonia and ventilator-associated bacterial pneumonia and for the treatment of infections due to aerobic Gram-negative organisms in adults with limited treatment options [2,3,4]. 

Cefiderocol approved dosage is 2 g administered every 8 h by intravenous infusion over 3 h. The dosage must be adjusted according to renal function as creatinine clearance (CrCL) was the most significant covariate in population pharmacokinetic studies [5,6]. Indeed, cefiderocol is primarily eliminated by the kidneys. Similar to other beta-lactam antibiotics, the cefiderocol efficacy target is the percentage of the dosing interval during which free drug concentrations are above the minimal inhibitory concentration (MIC) (%fT > MIC) [7]. Recommendations for optimal clinical response in intensive care patients are that residual plasma concentrations of beta-lactams antibiotics should be four to eight times the MIC [8,9].

Several studies have shown a high inter- and intra-individual variability of plasma concentrations of antibiotics, especially beta-lactam antibiotics in critically ill patients [10]. Pharmacokinetic (PK) analysis of cefiderocol has been described in healthy subjects and in patients with complicated urinary tract infections [5,6]. Cefiderocol is poorly metabolized and hepatic elimination represents a minor elimination pathway. Its protein binding, mainly to albumin, is around 40 to 60%. Cefiderocol terminal elimination half-life is about 2–3 h and its mean total and renal clearances in healthy volunteers were 5.46 L/h and 3.89 L/h, respectively [11,12]. However, data on cefiderocol PK properties in critically ill patients are currently lacking. This retrospective study was conducted to investigate individual characteristics that can influence cefiderocol pharmacokinetics in real life in order to optimize drug dosage.

## 2. Materials and Methods

### 2.1. Patients and Drug Assay

All patients included in this study were hospitalized in the intensive care units (ICU) at Pitié-Salpêtrière hospital. Patients were treated by cefiderocol in combination with other antibiotics for ventilator-associated bacterial pneumonia (VABP). As almost all patients were hospitalized in the ICU for more than a few days, all patients included in the study had ICU-induced malnutrition, as assessed by their low albumin level [13]. Blood samples were collected into lithium heparin tubes at steady state, one prior to the start of the infusion (Ctrough) and the others during and after the end of infusion. Blood samples were transferred to the laboratory within 2 h. Plasma samples were prepared by centrifuging collected blood samples for 5 min at 4500× *g* at 4 °C. All plasma samples were frozen at −80 °C until analysis. Plasma cefiderocol concentrations were assayed by an ultra-performance liquid chromatography system coupled with mass tandem spectrometry in a positive ionization mode (UPLC-MS/MS), as previously described by Llopis et al. [14]. Patients characteristics that could influence pharmacokinetics were collected retrospectively during the study. 

The fat-free mass (FFM) was determined after the equation [15]:

FFM = WHSmax·HT^2^·BW/(WHS50·HT^2^ + BW)

where WHSmax is the maximum FFM for a given height (HT, m) and WHS_50_ is the total bodyweight (BW, Kg) value when FFM is half of WHSmax. WHSmax is 42.92 and 37.99 kg/m^2^ and WHS50 is 30.93 and 35.98 kg/m^2^ for males and females, respectively. 

The CKD (Chronic Kidney Disease Epidemiology Collaboration) equation was used to estimate glomerular filtration rate. 

This retrospective study was based on data extracted from medical records and was performed in compliance with French regulations and according to the reference methodology MR-004, established by French National Commission on Informatics and Liberties (CNIL).

### 2.2. Data Analysis

Cefiderocol time-courses were fit to a two-compartment open model with first-order elimination. The following compartmental parameters were then derived: CL, Q, V1 and V2, which stand for the elimination and inter-compartmental clearances, central and peripheral volumes of distribution, respectively.

The nonlinear mixed effect modelling program Monolix version 2020R1 (Lixoft, Antony, France) (http://lixoft.com (accessed on 1 January 2022)) was used for the model development. The between-subject, BSV or ω and residual variabilities (square roots of the variances ω^2^ and σ^2^) were ascribed to an exponential distribution. The influence of demographic and clinical characteristics that could affect cefiderocol pharmacokinetics, i.e., sex, total bodyweight (BW), FFM, age and renal function (CKD equation) were investigated.

Because the drug is albumin-bound in plasma, the effect of albuminemia was also investigated after the following pharmacokinetic principles, i.e., the elimination and exchange processes are thought to depend upon the unbound drug concentration, C_U_, as:

C_B_(t) = C_U_(t) × K_BIND_ × ALB, non-saturable binding or

C_B_(t) = C_U_(t) × ALB/(C_U_(t) + Kd), saturable binding

dA1(t)/dt = R – CL_U_ × C_U_(t) − k_12_ × A_1_(t) + k_21_ × A_2_(t) where k_12_ = Q_U_/V_1U_ and k_21_ = Q_U_/V_2U_

dA_2_(t)/dt = k_12_ × A_1_(t) − k_21_ × A_2_(t)

C_1_(t) = C_U_(t) + C_B_(t)

where A_1_(t), A_2_(t) and R are the drug amounts in the 1st and 2nd compartments and infusion rate. The drug exchanges between compartments 1 (central) and 2 (peripheral) are driven by the transfer rate constants k_12_ and k_21_. C_B_(t) is the albumin-bound concentration assuming a non-saturable or saturable binding to albumin with K_BIND_, binding constant (L/g) or Kd, dissociation constant. The observed total plasma concentration is then fitted after the model predicted C_1_(t) and the corresponding clearance and volume terms are designed by the subscript U. Note that CL and V terms for total drug concentration kinetics are simply derived from CL = f_U_ × CL_U_ and V = f_U_ × V_U_ where f_U_ = 1/(1 + K_BIND_ × ALB).

The corrected Bayesian Information Criterion (cBIC) was used to test different hypotheses regarding the final model. The covariate sub-model was evaluated both via the BICc and BSV values. A covariate effect was finally retained, provided its effect could be physiologically explained. Each model was evaluated by visual inspection of goodness-of-fit plots, mainly observed-predicted (population and individual) concentration scatter plots. The normalized prediction distribution error metrics, whose mean and variance should not be different from 0 and 1 with a normal distribution, were preferred over the visual predictive checks (VPC), because the various inter-dose intervals rendered the latter difficult to interpret. Diagnostic graphics and other statistics were obtained using the R software.

## 3. Results

A total of 205 plasma samples were obtained from 55 patients, 37 males and 18 females. All patients were treated with cefiderocol for ventilator-associated pneumonia with carbapenem-resistant gram negative bacteria (GNB) including Pseudomonas aeruginosa (52), Stenotrophomonas maltophilia (2) and Acinetobacter baumanii (1). Patients’ characteristics are summarized in Table 1. The median of residual concentration (C0), and maximal concentration (Cmax) were 35 mg/L [range 19–67] and 60 mg/L [range 40–84] respectively.

There were no concentrations below the limit of quantification. Patients received cefiderocol as 750 to 2000 mg infusions every 6, 8 or 12 h. At the time of sampling, severe renal impairment (CKD < 30 mL/min/1.73 m^2^) was diagnosed in 10 (14.5%) patients. In 16 patients (29%) the value of CKD was greater than 120 mL/min/1.73 m^2^ (median 150 mL/min/1.73 m^2^ Interquartile range (IQR) (143–164).

The population parameters of the covariate-free model were satisfactorily estimated. Only BSV for CL and Q, ω_CL_ and ω_Q_, could be estimated. Table 2 summarizes the covariates sub-models tested (only the models that produced a cBIC value lower than the base model cBIC value are shown). CKD positively influenced CL and decreased both ω_CL_ and the cBIC value. The non-saturable albumin binding effect alone was also significant (saturable binding was unidentifiable and failed to converge). The K_BIND_ value was fixed to 0.036 L/g assuming a 41% free concentration fraction for a 40.5 g/L albuminemia [16]. When clearance and volume terms were related to size effects, WT or FFM, (SIZE/mean_SIZE_)^p_size^ (p_size exponents fixed to 0.75 and 1 for clearances and volumes according to the allometric rule), only the FFM effect was significant. The final model combined the CKD and albumin binding effects (the further addition of FFM did not significantly improve this model). A sensitivity analysis on K_BIND_ assuming f_U_ values of 50 and 60% (K_BIND_ = 0.025 or 0.017) provided cBIC slightly greater than that of the final model. The final covariate sub-model for CL_U_ was then:

CL_U_ (L/h) = 7.38 × (CKD/100)^0.426^

or, adding the FFM effect

CL_U_ (L/h) = 7.33 × (FFM/54)^0.75^ × (CKD/100)^0.416^

Parameter estimates are summarized in Table 3. Note that the shrinkages for CL_U_ and Q_U_ were 1.84 and 81%.

**Figure 1:**
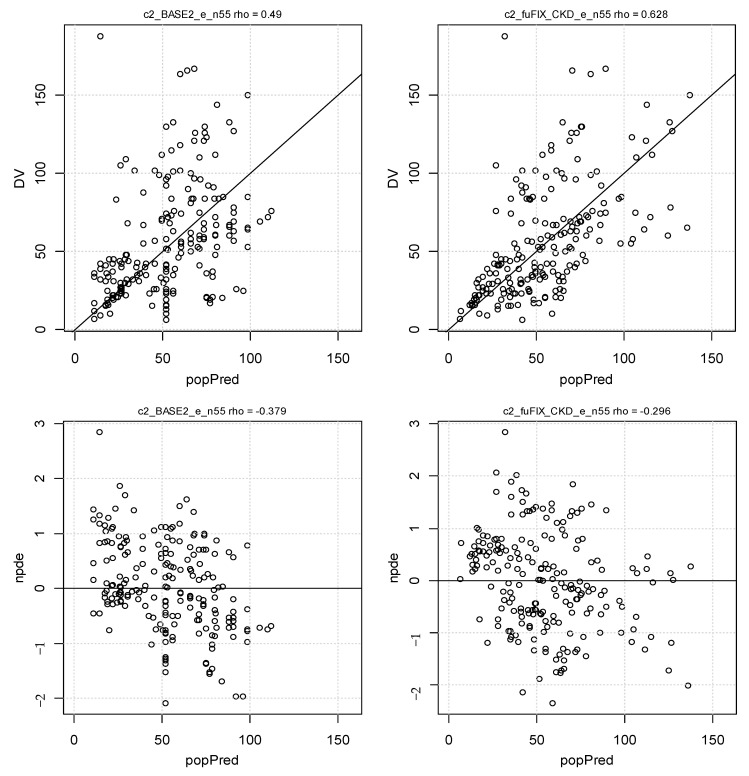
Goodness-of-fit plots for the base (covariate-free, **left**) and final (CKD + albumin binding, **right**) models. DV and popPred, observed and population predicted concentrations; npde, versus popPred values with the y = zero line.

**Figure 2:**
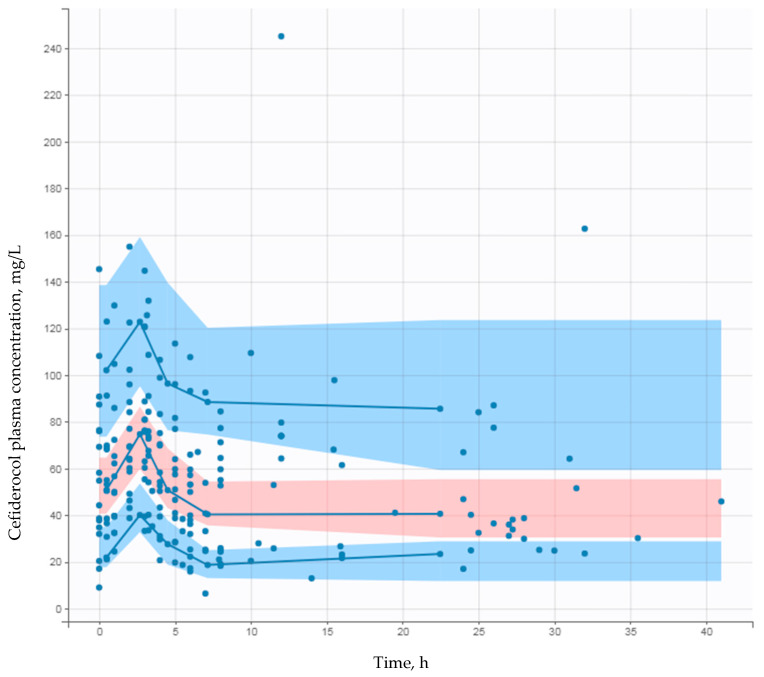
Prediction-corrected visual predictive check for the final cefiderocol population pharmacokinetic model. Plain (●) and blue lines stand for prediction-corrected observed concentrations and their 5th, 50th and 95th percentiles. Light blue and red bands stand for the corresponding model predicted 90% confidence intervals.

The goodness-of-fit plots for the final model shown in Figure 1 and Figure 2 show the visual predictive checks for the cefiderocol final PK model. The observed concentration percentiles are well included in the corresponding model-predicted 90% confidence interval bands. 

**Figure 3:**
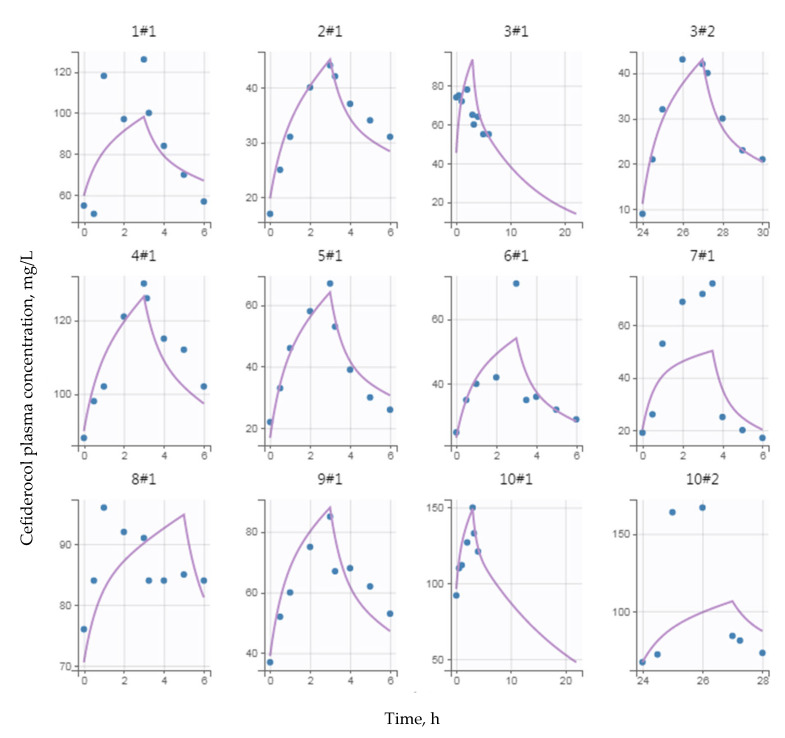
Representative individual curve fitting of cefiderocol (circles, observed concentration; purple solid lines, individual fits; N1#N2, *i*th subject # *i*th occasion).

Finally, Figure 3 depicts the model curve fittings for some individuals. The mean and standard deviation of the normalized prediction distribution errors (NPDE), −0.002 and 1.15, were not significantly different from 0 and 1 (*p* = 0.98 and *p* = 0.15) with a symmetrical distribution around 0, as expected for these metrics.

### Dosage Recommendations

**Figure 4:**
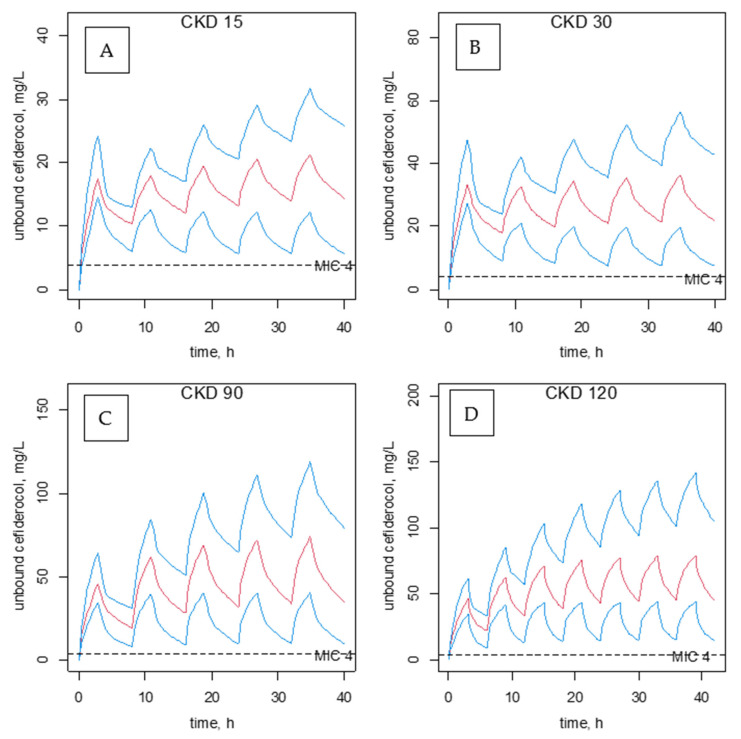
Unbound plasma cefiderocol concentration-time courses for 4 typical renal function (CKD = 15, CKD = 30, CKD = 90, CKD = 120), receiving doses of (1000 mg then 3 × 500 mg) panel (**A**), (2000 mg then 3 ×1000 mg) panel (**B**), (3 × 3000 mg) panel (**C**), (4 × 3000 mg) panel (**D**) by 3 h infusion, respectively. Doses are administered every 8 h except for CKD = 120, every 6 h. Note total plasma cefiderocol concentration is obtained by multiplying the unbound concentration by 2.5 assuming a 40 g/L albuminemia (f_U,40g/LALB_ = 0.6). The horizontal dashed line is drawn at 4 mg/L (considering the ELF-to-CU concentration ratio is 0.5) Blue lines stand for prediction concentrations and their 5th, and 95th percentiles. Red and blue lines represent means and 5th–95th percentiles for predicted concentrations, respectively.

Cefiderocol approved regimen is at a dose of 2 g every 8 h for the treatment of adult patients for whom treatment options are limited with a bacterial MIC ≤ 2 mg/L. Optimal clinical response of β-lactam antibiotics is obtained with a residual plasma concentration ≥ 4–8 times the MIC [8,9]. Positive clinical outcome was associated with increasing 100% fT > MIC ratio in infected critically ill patients [17]. The dose of cefiderocol can be directly estimated from the model-predicted unbound concentrations given the renal function index CKD. Figure 4 represents a proposal for the doses of cefiderocol to be administered according to various levels of renal function for a MIC value of 2 in situ, i.e., considering the epithelial lining fluid (ELF) where the ELF-to-C_U_ concentration ratio is (0.5) two hours post-end of infusion [18]. The probability of target attainment, in these intensive care patients with an undernutrition status, for > 99% fT > MIC was > 99% when MIC was 4 mg/L.

## 4. Discussion

To date, most of the pharmacokinetics parameters of cefiderocol have been generated from phase one, two and three clinical studies [19,20,21,22]. In this study, we showed that cefiderocol time-courses were well described by a two-compartment model. The limited number of patients, n = 55, allowed the estimation of only one between-subject variability parameter, ω_CL_. Kawaguchi et al. [6] described cefiderocol pharmacokinetics after using a three-compartment model. However, the volume of the central compartment, 0.73 L, was very small and can only be determined with a rich data sampling at early times post-infusion.

**Figure 5:**
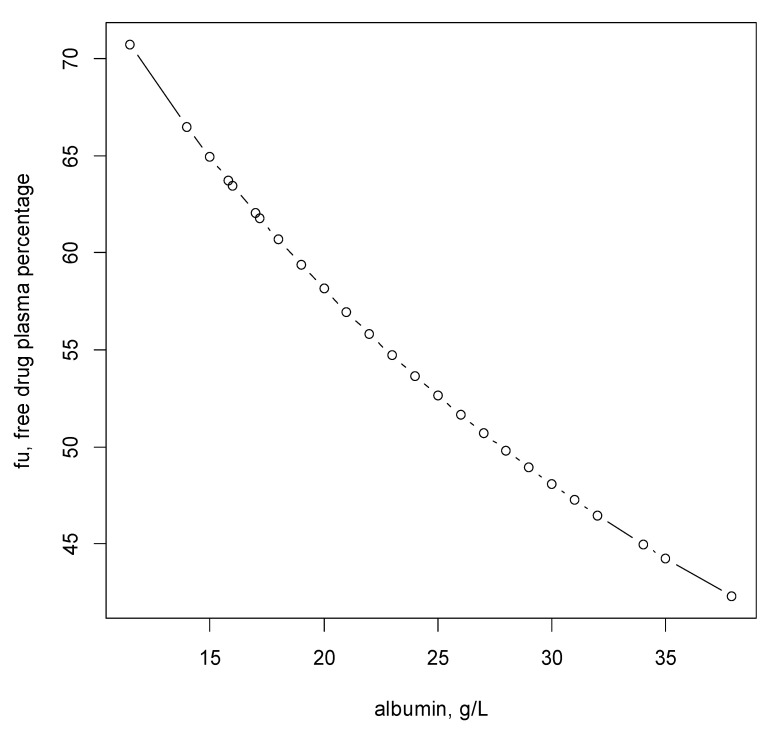
Relationship between f_U_ and the plasma albumin concentration.

The pharmacokinetic/pharmacodynamic (PK/PD) index of cefiderocol with bactericidal activity is correlated with fraction of time for which the free drug concentration in plasma exceeds the minimum inhibitory concentration of the infecting microorganism over the dosing interval (%fT > MIC) [5,7]. In this study of patient hospitalized in intensive care unit, albumin levels were low (median 22 g/L IQR (18–24)). In this modeling, the plasma albumin binding could be accounted for, thanks to the wide variation in albuminemia observed in these patients. The output of the pharmacokinetic model was ascribed to the unbound drug concentration which is thought to be the freely exchangeable drug in the body. The result was then fitted to the total drug concentration by adding the drug-bound concentration, based on albumin concentration. Compared to the same model based on total concentration, the cBIC value dropped by 16 units. Moreover, the relative precision of CKD effect on CL_U_ was 8.77%, as compared to 13.3% when based on total concentration. This demonstrates that the model based on the diffusible free concentration is more appropriate, which was expected for this renally-eliminated hydrophilic drug. Figure 5 shows that, for each patient, fu is determined as a function of its albuminemia. 

The K_BIND_ value was fixed according to the f_U_ values observed for various albumin concentrations, as previously reported [16]. This allowed the estimation of the unbound drug’s kinetic parameters. The CKD value was the main relevant covariate effect on the unbound drug clearance (CL_U_) that was not unexpected because of the very hydrophilic nature of cefiderocol (logP = −2.27). There was also an effect of FFM (but not total body weight) that was not unexpected given the hydrophilic nature of cefiderocol. However, this effect was not retained in the final model because it did not provide significant improvement. Nevertheless, in future studies, this FFM covariate should be considered instead of total body weight. The final model included the albumin-binding effect plus the renal function effect via the CKD index. Interestingly, the kinetic parameters for the unbound drug allow the prediction of the unbound, active, drug concentration, given the patient’s CKD is known. The CL value relative to the total drug concentration is 2.95 L/h (0.4 × 7.38). This low value, as compared to that reported by Kawaguchi et al. [6] (i.e., 4.2 to 5.0 L/h), may result from the poor condition of the patients, presenting various degrees of malnutrition.

Our study has some limitations. The small number of patients in our study did not allow an external validation of our model. In addition to be in an ICU, these patients exhibit a significant degree of undernutrition. Note that unbound concentrations were not measured, and that total and unbound concentration have been translated into each other simply by applying multiplicative constants. Moreover, MICs of cefiderocol were not available for all samples. Due to the shrinkage level observed on Q_U_, but not CL_U_, a pharmacokinetic-based individualization is not advised. In addition, it is usually accepted that ICU patients’ pathophysiologic state can rapidly change. New studies including ICU patients should be done for this recent antibiotic. Finally, estimates of renal function have low accuracy and precision in critically ill patients [23].

## 5. Conclusions

In conclusion, our study showed that albumin binding and CKD effects were significant predictors of unbound and total plasma cefiderocol concentrations. Our results indicate that individual adjustment of cefiderocol can be used to reach high minimum inhibitory concentrations based on an estimation of unbound drug concentration. Whether such an estimation results in a better therapeutic outcome remains to be determined.

## Figures and Tables

**Table 1:** Demographic and biological characteristics of the 55 patients (37 males/18 females).

	Mean	%RSD	Median	Min	Max
Age, years	55.5	30.3	60	14	80
Total bodyweight, Kg	79.9	20.5	76	29	120
Height, m	1.75	5.76	1.78	1.52	2.01
Body mass index, Kg/m^2^	26	18.5	26	25.5	39.8
Fat-free mass, Kg	55.6	17.9	53.6	23.6	79
Creatinine, µmol/L	132	113	86	17	736
CKD, mL/min/1.73 m^2^	79.6	61	70	6	170
Albumin, g/L	22.6	29	22	11.5	37.9
Plasma proteins, g/L	55.6	17.3	57	37	77
ASAT, U/L	39.5	79.9	28	12	183
ALAT, U/L	36.4	137	21	6	208
CRP, mg/L	84.7	78.2	87	1	302

Abbreviations: ALAT: alanine aminotransferase, ASAT: aspartate aminotransferase, CRP: C-reactive protein, CKD (Chronic Kidney Disease Epidemiology Collaboration) %RSD: Relative standard deviation in %. All data were collected at the time of cefiderocol sampling.

**Table 2:** Covariate sub-model building.

Model	Covariate	Parameter	Estimate	ω	cBIC
6.	^#^FREE + CKD	CL_U_	7.38	0.467	1682
5.	^#^FREE + CKD + FFM	CL_U_	7.33	0.464	1686
4.	^#^FREE + CKD + WT	CL_U_	6.84	0.491	1689
3. FFM	FFM-based allometry	CL	4.03	0.459	1698
2. CKD	Effect of CKD on CL	CL	4.07	0.461	1698
1. FREE ^#^	albumin binding, C_T_ = C_U_ + C_B_	CL_U_	6.26	0.627	1726
0	covariate free, basic	CL	3.47	0.634	1732

Abbreviations: CL or CLU, clearance or unbound drug clearance; ω, square root of between-subject variance ω^2^, for CL or CLU; FFM, fat-free mass (Kg); WT, total bodyweight (Kg); CKD (mL/min/1.73 m^2^), renal function index; cBIC, corrected Bayesian Information Criteria; CT, CU or CB, total, unbound or bound concentration; ^#^FREE, model taking into account the drug binding to albumin then estimating the unbound drug pharmacokinetic parameters, CL_U_, V1_U_, etc., related to the unbound drug concentration.

**Table 3:** Cefiderocol population parameters estimates for the unbound drug pharmacokinetics from 205 total plasma concentrations in 55 adult patients.

**Population Parameters**	**Estimate**	**RSE (%)**
V1_U_, L	17	22
CL_U_, L/h per CKD = 100	7.38	6.8
Q_U_, L/h	34	39
V2_U_, L	46	12
CKD effect on CL_U_	0.426	8.8
K_BIND_, FIXED (albumin binding constant), L/g	0.036	NA
**Statistical Parameters**		
ωCLu	0.467	10.4
ωQu	0.706	44
log-additive residual variability	0.183	6

Abbreviations: RSE, relative standard error in %; ω, square root of between-subject variance ω^2^; CKD (mL/min/1.73 m^2^), renal function index; Clu = 7.38 × (CKD/100)^0.426^; f_U_ = 1/(1 + ALB * 0.036) with f_U_ and ALB unbound drug fraction and albumin concentration in L/g. Note that CL and V terms for total drug concentration kinetics are simply CL = f_U_ × CL_U_ and V = f_U_ × V_U_. The total concentration is C_T_ = C_U_ × (1 + K_BIND_ × ALB) with C_U_ = f(Rate, CL_U_, V1_U_, Q_U_, V2_U_).

## Data Availability

The data support the findings of this study are available on request from the corresponding author upon reasonable request.
